# An Ectopic Imaging Window for Intravital Imaging of Engineered Bone Tissue

**DOI:** 10.1002/jbm4.10028

**Published:** 2018-01-31

**Authors:** Pieter‐Jan Stiers, Nick van Gastel, Karen Moermans, Ingrid Stockmans, Geert Carmeliet

**Affiliations:** ^1^ Laboratory of Clinical and Experimental Endocrinology Department of Chronic Diseases, Metabolism and Ageing KU Leuven Leuven Belgium; ^2^ Prometheus Division of Skeletal Tissue Engineering KU Leuven Leuven Belgium

**Keywords:** ANALYSIS/QUANTITATION OF BONE, BIOENGINEERING, IMPLANTS

## Abstract

Tissue engineering is a promising branch of regenerative medicine, but its clinical application remains limited because thorough knowledge of the in vivo repair processes in these engineered implants is limited. Common techniques to study the different phases of bone repair in mice are destructive and thus not optimal to gain insight into the dynamics of this process. Instead, multiphoton‐intravital microscopy (MP‐IVM) allows visualization of (sub)cellular processes at high resolution and frequency over extended periods of time when combined with an imaging window that permits optical access to implants in vivo. In this study, we have developed and validated an ectopic imaging window that can be placed over a tissue‐engineered construct implanted in mice. This approach did not interfere with the biological processes of bone regeneration taking place in these implants, as evidenced by histological and micro–computed tomography (μCT)‐based comparison to control ectopic implants. The ectopic imaging window permitted tracking of individual cells over several days in vivo. Furthermore, the use of fluorescent reporters allowed visualization of the onset of angiogenesis and osteogenesis in these constructs. Taken together, this novel imaging window will facilitate further analysis of the spatiotemporal regulation of cellular processes in bone tissue–engineered implants and provides a powerful tool to enhance the therapeutic potential of bone tissue engineering. © 2017 The Authors *JBMR Plus* published by Wiley Periodicals, Inc. on behalf of American Society for Bone and Mineral Research.

## Introduction

Over the past decades, tissue engineering has made considerable progress in the regeneration and even replacement of diseased and injured tissue in preclinical models.^1^ However, translation into clinically relevant applications has remained limited, in part due to our incomplete understanding of the in vivo mechanisms driving regeneration in these engineered tissues. Bone tissue engineering also faces similar problems. This discipline aims to mimic the complex and dynamic interplay between the different cell types that control successful bone repair,^2^, ^3^ but knowledge of these processes at high temporal and spatial resolution is lacking.^4^ Techniques such as histology and ex vivo micro–computed tomography (μCT) provide information at (sub)cellular level, but are destructive. They therefore require a significant number of replicates to allow inference on the dynamics of bone repair. Bioluminescence permits the longitudinal follow‐up of single samples, but the imaging resolution does not allow detailed visualization of different cell populations or cellular processes. Enhanced insight into the processes that contribute to successful repair therefore requires a method for nondestructive, high‐resolution visualization of bone tissue–engineered constructs in vivo. A promising approach for the study of living tissue exists in the combination of intravital microscopy with multiphoton fluorescence excitation, which is increasingly being applied to investigate tumor biology and host reactions on implanted biomaterials.^5^, ^6^

Multiphoton‐intravital microscopy (MP‐IVM) relies on the nonlinear excitation of fluorophores through absorption of two or more photons at once.^7^ This means that near‐infrared excitation wavelengths can be used which penetrate deeper into the tissue, result in less off‐target phototoxicity, and allow the label‐free visualization of collagen fibrils through second‐harmonics generation.^8^, ^9^ This technique is therefore well suited for minimally‐invasive 3D visualization of living tissues. However, any tissue between the microscope objective and the region of interest such as skin or fibrous tissue causes significant scattering of photons, and a clear optical path to the tissue or organ of interest is therefore recommended.^10^ This problem is often overcome by surgically exposing the tissue of interest, either only once during a short period or alternatively, in a repetitive way allowing more prolonged dynamic evaluation.^11^, ^12^ This second approach places significant stress on the animal and limits the frequency and total number of imaging sessions to less than once per week.

An alternative approach is to expose the tissue to be visualized under an imaging window shielded from the environment by optical glass.^5^, ^13^, ^14^, ^15^ This strategy permits recurrent visual access, eliminating the need for repeated surgery and allowing longer and/or more frequent MP‐IVM experiments. Indeed, intravital imaging is used to perform long‐term skeletal lineage tracing^16^, ^17^ and to visualize angiogenesis during bone repair,^11^, ^18^, ^19^ but without an imaging window, significant time for recovery from the surgery is needed between visualizations. The development of an imaging window that allows visualization of the early phases of bone regeneration would therefore be of great benefit.

In this study, we have developed and validated an imaging window that can be placed over an implanted construct in mice and allows MP‐IVM of implanted bone tissue‐engineered constructs at high resolution and frequency over extended periods without influencing the bone regeneration process. This new imaging window permits tracking of individual implanted cells during the first days of implantation, as well as visualization of host‐derived vascularization of the construct and the onset of osteogenesis.

## Materials and Methods

### Mice

The following strains of mice were used at 8 weeks of age: female wild‐type C57Bl/6 mice (R. Janvier Breeding Center, Saint‐Berthevin, France), female mice constitutively expressing enhanced green fluorescent protein (eGFP; B6.Cg‐Tg(ACTb‐eGFP))^20^ or conditionally expressing DsRed under control of the collagen type I promoter (B6.Tg(Col1a1‐cre/ERT2,‐DsRed)1Smkm).^21^ All mice were kept in non–specific pathogen‐free housing at 22°C, fed normal chow diet (V1535; Ssniff GmbH, Soest, Germany) and maintained in a 14‐hour light/10‐hour dark cycle. All animal experiments were conducted according to the regulations and with approval of the Animal Ethics Committee of the KU Leuven.

### Cell cultures

Murine periosteum‐derived cells (mPDCs) were obtained as described.^22^ Briefly, tibias and femurs were dissected and cleaned, their epiphyses were embedded in 5% low melting point agarose (SeaPlaque; Lonza, Verviers, Belgium) and periosteal cells were released from the periosteal surface by two collagenase‐dispase digests. The first digest (10 min) was discarded, the second digest (50 min) was washed, pelleted, and seeded. Cells were cultured in αMEM with 2mM GlutaMAX‐I, supplemented with 10% fetal bovine serum, 100 U/mL penicillin and 50 μg/mL streptomycin (all from Gibco, Life Technologies, Ghent, Belgium). For experiments with fibroblast growth factor 2‐pretreated mPDCs (mPDC^FGF2^), the culture medium was supplemented with 5 U/mL heparin (LEO Pharma, Ballerup, Denmark) and 5 ng/mL human recombinant FGF2 (R&D Systems, Minneapolis, MN, USA) from isolation until the cells were used for experiments, mostly after 3 weeks.

### Scaffold implantations

Two different types of scaffolds were used. In the first approach, mPDCs of passage 3 were seeded onto calcium/phosphate‐collagen–based scaffolds. More precisely, NuOss Collagen scaffolds (ACE Surgical Supply Company, Brockton, MA, USA) of 3 × 3 × 3 mm^3^ seeded with 1 × 10^6^ cells per scaffold at a density of 5 × 10^7^ cells per mL were used, or CopiOs Bone Void Filler (Zimmer Spine, Minneapolis, MN, USA) of 3 × 3 × 2.5 mm^3^ with 8 × 10^5^ cells per scaffold seeded at a density of 4 × 10^7^ cells per mL. Seeded scaffolds were incubated overnight without agitation and implanted subcutaneously on the back (two per mouse as described^23^) or in an imaging window as further described. Second, as a model of endochondral bone formation, mPDC^FGF2^ of passage 3 were resuspended in collagen type I (5 mg/mL in PBS; Corning GmbH, Wiesbaden, Germany) at a density of 1 × 10^7^ cells per mL, and 100 μL was implanted subcutaneously as described.^24^

### Imaging window surgery

Two types of imaging window were evaluated. First, the dorsal skinfold window chamber (APJ Trading Co, Inc, Ventura, CA, USA) was surgically prepared following a published protocol.^25^ Briefly, mice were anaesthetized with ketamine (100 mg/kg; Nimatek; Eurovet Animal Health B.V., Bladel, The Netherlands) and xylazine (15 mg/kg; XYL‐M; VMD N.V., Arendonk, Belgium), depilated, and a skinfold was created on the back between two titanium frames. The cutis and subcutis were removed on one side of the skinfold and sealed with a coverslip, creating a protected subcutaneous chamber into which samples can be implanted and from which they can be imaged. Animals were left to recover for 48 hours after surgery, at which time the chamber was reopened, then a scaffold or collagen gel was inserted, followed by closing with a new coverslip, corresponding to day 0 of implantation.

Second, an in‐house ectopic imaging window was developed based on a recently published design.^26^ Briefly, the window consisted of a stainless‐steel ring with a large groove running along the outside, and a small rim on the inside onto which an 8‐mm coverslip was glued using cyanoacrylate (Histoacryl; B. Braun, Melsungen, Germany). When using this ectopic window, the cell‐seeded construct was first implanted subcutaneously on the back of the animal. After 24 hours of recovery, an incision was made over the implanted construct and the imaging window was fixed into this incision by bringing the skin tightly into the groove of the window using a purse‐string suture.

### MP‐IVM

For sequential in vivo imaging experiments, mice were anaesthetized through inhalation of 2% isoflurane in O_2_. They were placed on a custom‐built stage containing a heating pad (Tecnilab‐BMI, Someren, The Netherlands), onto which the fixator for the imaging windows was attached. For visualization of the host vasculature, 100 μL of 10 mg/mL FITC‐dextran (70 kDa; Sigma‐Aldrich, Diegem, Belgium) in saline was injected via the tail vein just before the experiment. Intravital imaging was performed with a TCS SP8‐MP microscope (Leica Microsystems GmbH, Wetzlar, Germany) using a HCX IRAPO L 25×/0.95 objective immersed in water and a Chameleon Ultra II Ti:Sapphire laser (Coherent Inc., Santa Clara, CA, USA) tuned to 920 nm. At this wavelength, eGFP, FITC, and DsRed undergo excitation and collagen second‐harmonic generation (SHG) occurs at 460 nm. Imaging positions were retraced during subsequent visualizations using fiducial markers applied with a marker pen on the imaging window to assist with stage alignment and collagen structures detected by SHG. Emitted light was captured with descanned HyD detectors (bandpass filters for SHG: 455–465 nm, eGFP/FITC: 500–520 nm, DsRed: 560–640 nm) or non‐descanned HyD detectors (bandpass filters for SHG: 400–490 nm, eGFP/FITC: 500–540 nm, DsRed: 572–647 nm). Multiphoton images were captured at a pixel (voxel) size of 0.44 × 0.44 (×2) μm. Image stacks were merged using maximal intensity projection and tiled images were stitched using ImageJ v1.49 software (NIH, Bethesda, MD, USA; https://imagej.nih.gov/ij/),^27^ or using LAS AF 3.2 software (Leica Microsystems). Sequential images were aligned using SHG from extracellular matrix with custom‐made MeVisLab software (MeVis Medical Solutions AG, Bremen, Germany).

### Immunostaining and histochemistry

Following intravital imaging experiments or control ectopic implantation, constructs were recovered and fixed overnight in 2% paraformaldehyde at 4°C. Samples were decalcified in EDTA for 14 days at 4°C, embedded in NEG‐50 frozen section medium (Richard‐Allen Scientific, Thermo Fisher Scientific, Kalamazoo, MI, USA), and sectioned with a HM560 cryostat at 7 μm using SEC35 disposable steel blades (both from Thermo Fisher Scientific). Sections were either counterstained with Hoechst, stained with hematoxylin and eosin (H&E), or stained for tartrate‐resistant acid phosphatase (TRAP) visualizing osteoclasts, CD31 visualizing endothelial cells, or collagen type II using routine published protocols.^28^, ^29^, ^30^ CD31 staining was followed by H&E counterstain. Antibodies used for immunostaining are listed in Supporting Table 1. Images were taken on an Axioplan 2 light microscope (Carl Zeiss Microscopy GmbH, Jena, Germany) using a Plan‐Neofluar 20 × /0.5 objective.

### μCT

Radiographic images to detect mineralized tissue in in vivo implanted scaffolds were acquired using a SkyScan 1076 in vivo μCT (Bruker microCT, Kontich, Belgium). Upon sample retrieval and fixation, constructs were scanned using a SkyScan 1172 high‐resolution μCT at a pixel size of 5 μm with a tube voltage of 60 kV and a 0.5 mm aluminum filter. Reconstruction was performed with NRecon v1.6.8 software and mineralized tissue was quantified with CTAn v1.13 software (both from Bruker microCT) using a threshold value of 75 or using custom‐made MeVisLab software. The latter software enabled us to distinguish newly formed bone from scaffold granules based on grayscale values (MeVis Medical Solutions AG, Bremen, Germany).^24^

### Statistical analysis

Unless indicated otherwise, data are presented as mean ± SE. Littermates were randomly assigned to experimental conditions. Sample sizes were based on previous experiments showing significant effects on bone formation and comparable studies in literature. Each experiment was repeated at least twice. Researchers were blinded during data collection and analysis, such as μCT and histological analysis. Data were analyzed by two‐sided Student's *t* test using NCSS statistical software (NCSS LLC, Kaysville, UT, USA). Differences between groups were considered statistically significant at *p* < 0.05.

## Results

### mPDC^FGF2^ rapidly induce endochondral ossification upon ectopic implantation

**Figure 1:**
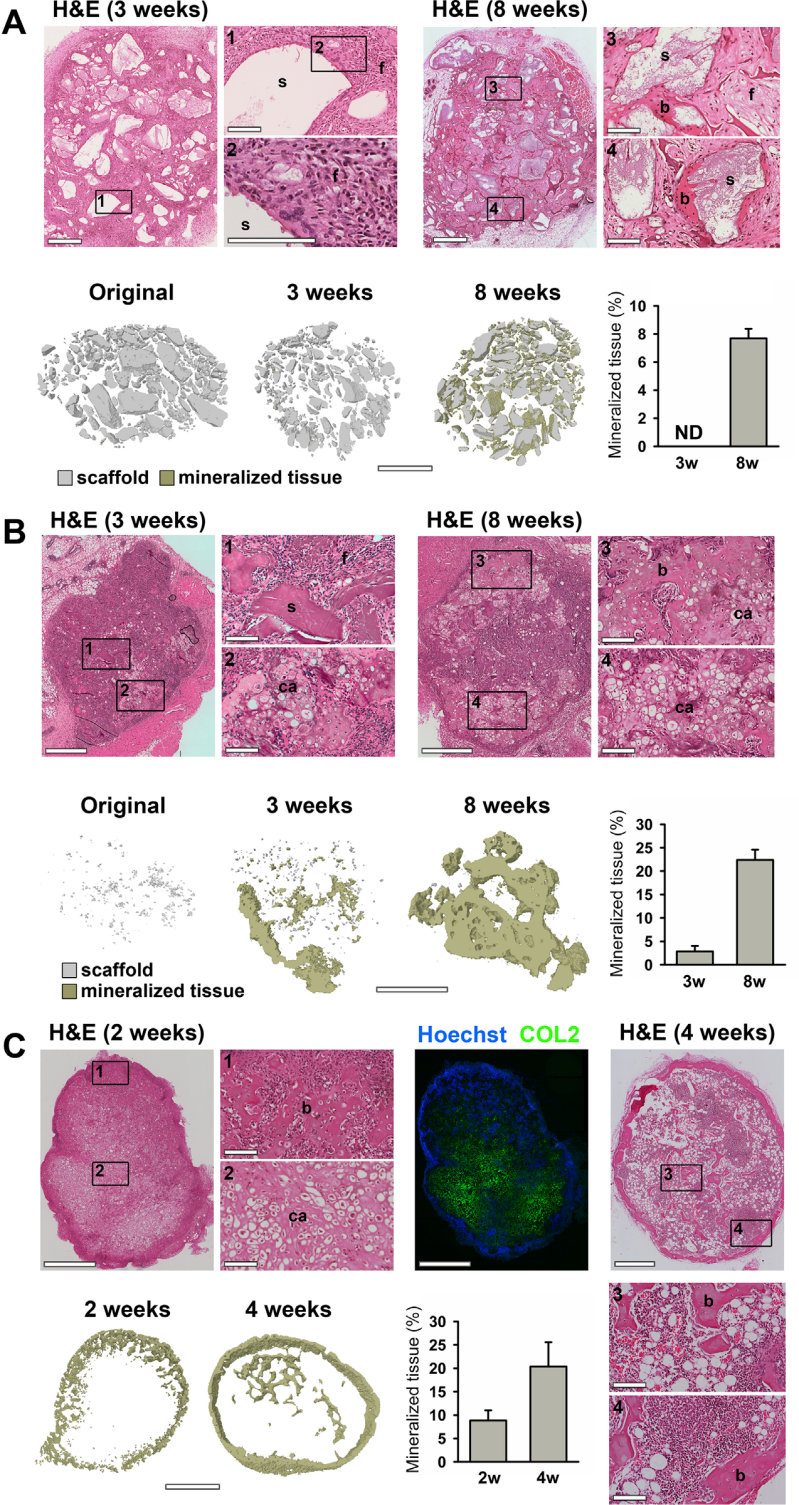
Evaluation of bone formation at early time points in different scaffolds. (*A*) H&E stainings (top) and μCT analysis and quantification of newly formed mineralized tissue (bottom) of NuOss scaffolds seeded with mPDC and implanted for 3 or 8 weeks (*n* = 4). A reference image of an unseeded scaffold that was not implanted is also shown (original). (*B*) H&E stainings (top) and μCT analysis and quantification of newly formed mineralized tissue (bottom) of CopiOs scaffolds seeded with mPDC and implanted for 3 or 8 weeks (*n* = 4). A reference image of an unseeded scaffold that was not implanted is also shown (original). (*C*) H&E stainings and collagen type II (COL2) immunostaining with Hoechst nuclear counterstain (top), and μCT analysis (bottom) and quantification of mineralized tissue of mPDC^FGF2^ suspended in a collagen gel and implanted for 2 or 4 weeks (*n* = 4–6). Scale bars = 1 mm (μCT), 500 μm (H&E), or 100 μm (details); s = scaffold; f = fibrous tissue; ca = cartilage; b = bone; ND = not detected.

Experiments using imaging windows such as the dorsal skinfold chamber often lead to loss of skin integrity, thereby limiting the possibility for in vivo imaging to a period of 3 weeks.^13^ Therefore, the important phases of bone regeneration in a tissue‐engineered construct have to occur within this timeframe to allow intravital imaging of this process in an imaging window. Considering these constraints, we first determined which model is best suited for implantation in an imaging window by evaluating the induction of bone regeneration in several scaffold types. NuOss scaffolds consist of a bovine collagen type I sponge mixed with relatively large (>100 μm) bovine cortical bone granules. Seeding these scaffolds with mPDCs and implanting them subcutaneously for 8 weeks resulted in new bone formation surrounding the scaffold granules, as detected by μCT and histological analysis (Fig. 1 *A*). However, new bone tissue was not yet detectable within the first 3 weeks of implantation, although the scaffolds were highly cellularized (Fig. 1 *A*). These data indicate that when using this scaffold only part of the bone formation process can be analyzed within the timeframe that is achievable in an imaging window. CopiOs Bone Void Filler is an alternative bovine collagen type I sponge mixed with small synthetic calcium phosphate granules. When this scaffold was seeded with mPDCs and implanted subcutaneously for 3 weeks, cartilage and mineralized tissue was formed (Fig. 1 *B*), but only in very localized regions and to a very limited extent compared to the amount formed after 8 weeks (Fig. 1 *B*). The scattered low amounts of newly formed cartilage tissue in this scaffold at early time points after implantation will impede efficient observation of bone regeneration during MP‐IVM because only a few locations and not the entire scaffold can be imaged in each session. We therefore analyzed the feasibility of a third described model.^24^ Pretreating mPDCs with FGF2 and implanting them in a collagen type I matrix subcutaneously resulted in bone formation via the endochondral ossification pathway. Indeed, a cartilage template is formed uniformly after 2 weeks, as evidenced by collagen type II staining (Fig. 1 *C*), and this is replaced by mature bone within 4 weeks postimplantation. The important phases of bone formation thus occur within 3 weeks and we therefore chose this model for validation of the imaging window for MP‐IVM.

### Validation of the dorsal skinfold window

**Figure 2:**
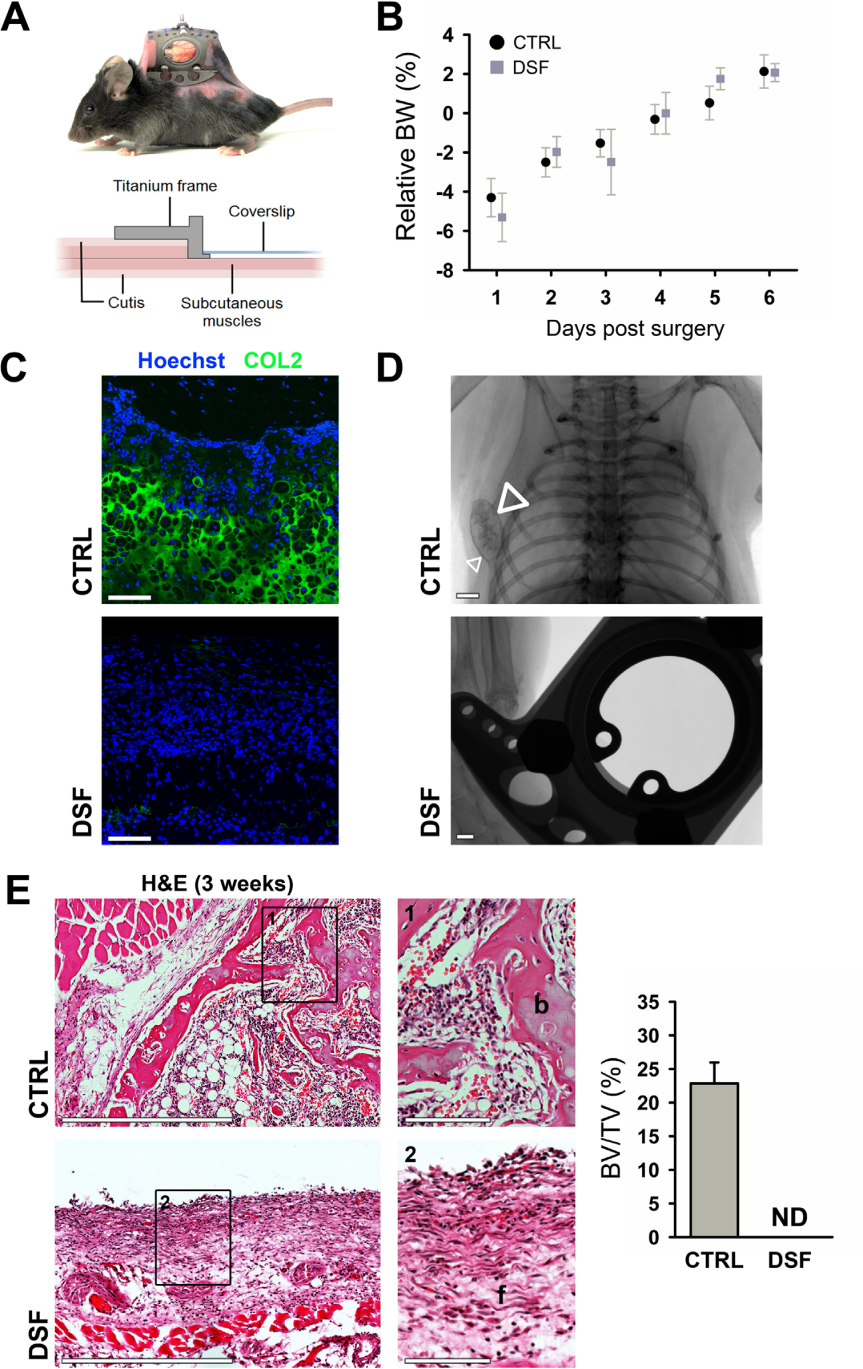
The dorsal skinfold window (DSF) negatively affects bone regeneration. (*A*) Pictorial and schematic overview of the implanted DSF. (*B*) Body weight recovery of mice receiving a DSF or CTRL ectopic implantation (*n* = 8). (*C*) COL2 immunostaining and Hoechst nuclear counterstain of mPDC^FGF2^‐seeded constructs implanted ectopically directly under the dorsal skin or in the DSF for 2 weeks. Scale bars = 100 μm. (*D*) Radiography of mPDC^FGF2^‐seeded collagen gels implanted ectopically or in the DSF for 3 weeks. Scale bars = 1 mm; arrowheads: mineralized construct. (*E*) H&E stainings of mPDC^FGF2^‐seeded collagen gels implanted ectopically or in the DSF for 3 weeks, and quantification of the amount of bone formed. Scale bars = 500 μm or 100 μm (details); *n* = 4. DSF = dorsal skinfold window; CTRL = control; COL2 = collagen type II; ND = not detected; b = bone; f = fibrous tissue.

We next investigated whether the dorsal skinfold imaging window was well tolerated and whether bone formation in the implanted scaffold occurred at the same pace when placed in the imaging window. The dorsal skinfold window (Fig. 2 *A*) has been used in combination with intravital microscopy to study the reaction of host tissues on implanted biomaterials,^6^, ^13^ but has not yet been evaluated to visualize bone formation in cell‐seeded implants. Mice that received a dorsal skinfold window showed normal behavior within 24 hours after surgery and their body weight recovered at a comparable rate to those that received an ectopically implanted construct (Fig. 2 *B*). After allowing the animals to recover for 48 hours, an implant containing mPDC^FGF2^ was placed inside the dorsal skinfold chamber. These constructs were subsequently recovered at indicated time points and compared to control ectopic implants. Control samples contained abundant cartilaginous collagen type II–positive matrix after 1 week, which was, however, not observed in dorsal skinfold samples, although numerous cells were still present (Fig. 2 *C*). Constructs implanted ectopically also formed mineralized tissue after 2 and 3 weeks as detected on radiographic images, whereas no mineralization was observed in the dorsal skinfold even after 3 weeks (Fig. 2 *D*). Histological examination of constructs implanted for 3 weeks showed manifest new bone formation, up to 23% of total tissue volume, in the control samples whereas only fibrous tissue was found in dorsal skinfold implants (Fig. 2 *E*). Taken together, these results indicate that the dorsal skinfold window does not provide an adequate physiological environment for endochondral bone formation by implanted mPDC^FGF2^, which prompted us to develop a new imaging window for MP‐IVM of bone tissue–engineered constructs.

### Validation of an ectopic imaging window

**Figure 3:**
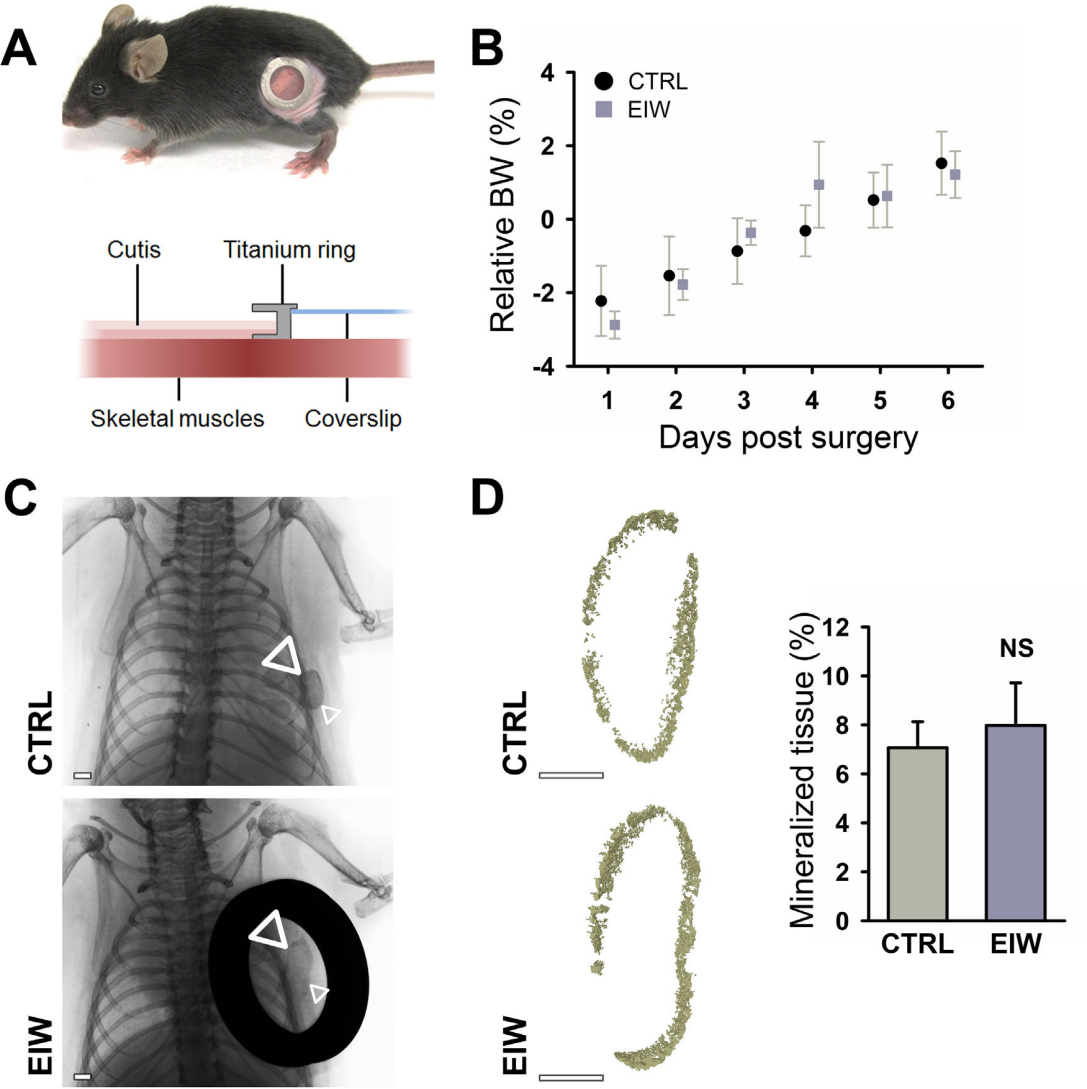
Validation of the ectopic imaging window (EIW). (*A*) Pictorial and schematic overview of the implanted EIW. (*B*) Body weight recovery of mice receiving an EIW or CTRL ectopic implantation (*n* = 6). (*C*) Radiography of mPDC^FGF2^‐seeded collagen gels implanted ectopically or exposed through an EIW for 2 weeks. Scale bars = 1 mm; arrowheads: mineralized construct. (*D*) μCT analysis of mineralized tissue in mPDC^FGF2^‐seeded collagen gels after ectopic or EIW implantation for 2 weeks, and quantification of the amount of mineralized tissue formed. Scale bars = 1 mm; *n* = 3. EIW = ectopic imaging window; CTRL = control; NS = not significant.

As an alternative to the dorsal skinfold window, we developed a smaller ectopic imaging window. This imaging window consists of a metal ring with a coverslip inside that can be placed over an implanted construct on the abdomen or flank of a mouse (Fig. 3 *A*). Because this window is less invasive and requires less time to implant, mice recovered very well from the surgery (Fig. 3 *B*). To investigate whether mPDC^FGF2^ could induce endochondral ossification in the ectopic imaging window, constructs were implanted subcutaneously and 24 hours later the overlying skin was resected followed by insertion of the ectopic imaging window. Radiography at 14 days after implantation showed mineralized tissue in control constructs as well as in those placed under the imaging window (Fig. 3 *C*). Samples were recovered and analyzed in detail using ex vivo μCT, which revealed that equal amounts of mineralized tissue had formed in both conditions (Fig. 3 *D*). These results indicate that the initial stages of endochondral ossification are not altered when mPDC^FGF2^ are implanted under the ectopic imaging window compared to the control ectopic assay.

### Endochondral ossification progresses normally in the ectopic imaging window

**Figure 4:**
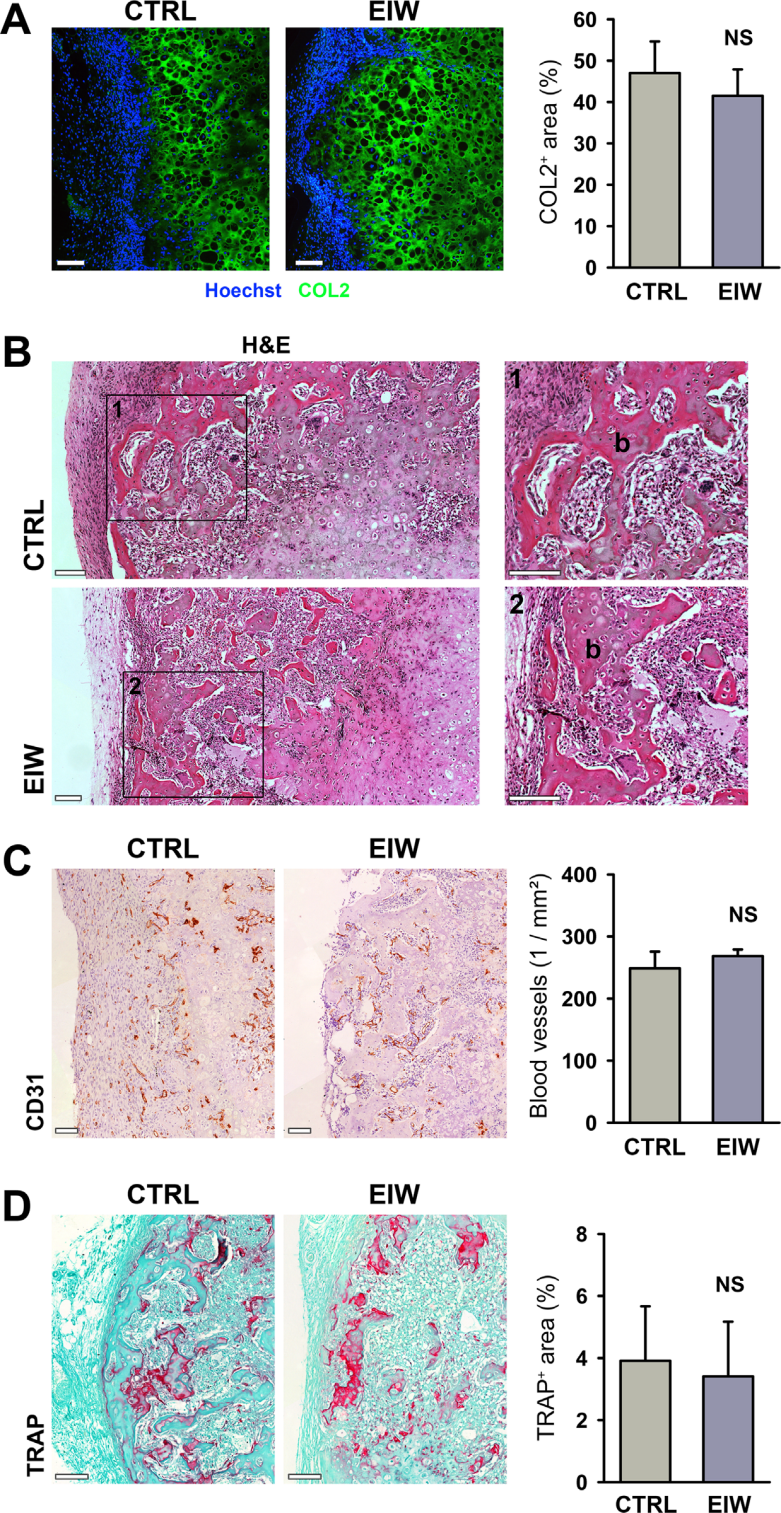
Endochondral ossification progresses normally in the EIW. (*A*) COL2 immunostaining and Hoechst nuclear counterstain of constructs containing mPDC^FGF2^ and implanted ectopically directly under the dorsal skin (CTRL) or in the EIW for 1 week, and quantification of the COL2‐positive area in these explants. Scale bars = 100 μm; *n* = 3. (*B*) H&E staining of mPDC^FGF2^‐seeded constructs, implanted ectopically or in an EIW for 3 weeks. Scale bars = 100 μm. (*C*) CD31 staining of mPDC^FGF2^‐seeded collagen gels implanted ectopically or in an EIW for 3 weeks and quantification of blood vessels in these constructs. Scale bars = 100 μm; *n* = 3. (*D*) TRAP staining of mPDC^FGF2^‐seeded collagen gels implanted ectopically or in an EIW for 3 weeks and quantification of the TRAP‐positive area in these constructs. Scale bars = 100 μm; *n* = 3. EIW = ectopic imaging window; COL2 = collagen type II; CTRL = control; NS = not significant; b = bone.

Next, we ensured that the progress of bone formation in the ectopic imaging window was comparable to the control assay at early and late time points, using the setup described in the paragraph above and analyzing the samples by histology. Collagen type II immunostaining showed that 1 week after implantation statistically comparable amounts of cartilage matrix had been formed in the two conditions (Fig. 4 *A*). H&E staining revealed that bone tissue formed through endochondral ossification was detectable in comparable amounts in both conditions after 3 weeks (Fig. 4 *B*). Vascularization of the implant at this time point was quantified on CD31‐stained sections and found to be similar in control and imaging window samples (Fig. 4 *C*). To confirm the presence of mature bone with remodeling activity, TRAP staining was performed and quantified. This analysis indicated that bone formation in control and imaging window samples had progressed similarly (Fig. 4 *D*). Together, these results show that the imaging window has no manifest effect on the process of endochondral ossification observed using implanted mPDC^FGF2^, highlighting the biological relevance of observations made via MP‐IVM in this model.

### MP‐IVM reveals implanted cell behavior and host interactions at high spatiotemporal resolution

**Figure 5:**
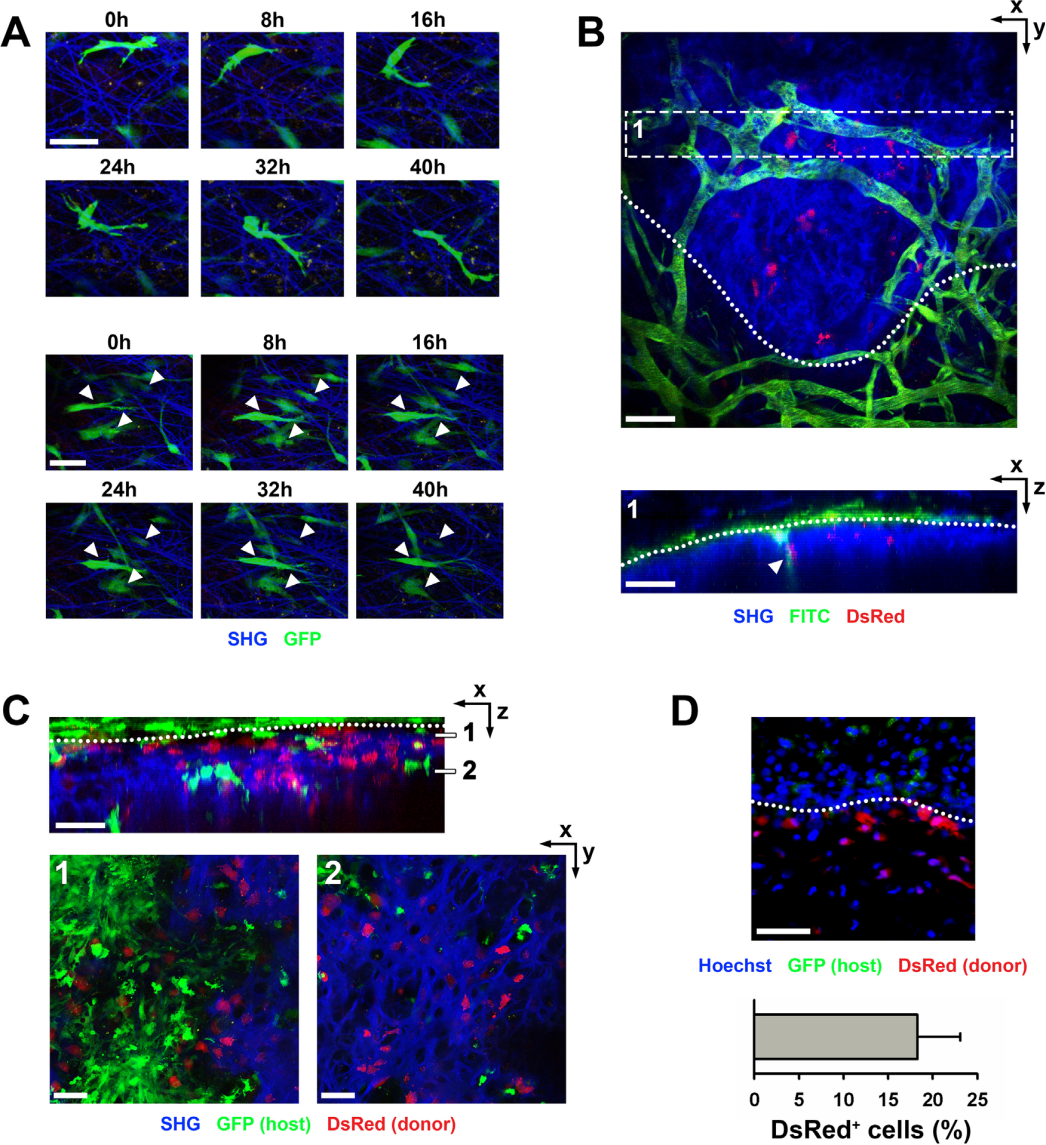
Serial multiphoton‐intravital imaging of mPDC^FGF2^‐seeded collagen gels implanted in the ectopic imaging window. (*A*) Tracing of single eGFP‐labeled cells, imaged every 8 hours starting at 24 hours after implantation. Some cells displayed clear polarization and migration (top panels), whereas most are stationary in relation to the matrix (bottom panels, white arrowheads). SHG derived from collagen fibers was used to align images. Images are maximum intensity projections of 68 slices. Scale bars = 20 μm. (*B*) Intravital visualization using fluorescent dextran (FITC) of host vasculature growing into the construct (white arrowhead) at day 10 after implantation. (Top panel) Maximum intensity projection of 82 slices. (Bottom panel) Dashed box resliced and shown in the depth axis. Scale bars = 100 μm. (*C*) mPDC^FGF2^ expressing a collagen type I reporter (DsRed), which labels early osteogenic cells, were implanted and visualized by intravital microscopy at day 10. Host cells express actin‐eGFP. (Top panel) Resliced image stack shown in the depth axis (79 slices). (Bottom panels) Maximum intensity projections of 6 slices taken at the depths indicated by dashes in the top panel. Scale bar = 100 μm. (*D*) Implants from *C* were recovered for histological analysis after intravital imaging. Expression of the collagen type I reporter (DsRed) was quantified in the implanted cells Scale bar = 100 μm; *n* = 4.

Finally, we evaluated the feasibility of repeated imaging of bone tissue–engineered constructs implanted in an ectopic imaging window. To this end, a mixed population of 90% wild‐type and 10% actin‐eGFP mPDC^FGF2^ was implanted, an ectopic imaging window was placed over the construct, and MP‐IVM was performed every 8 hours. This ratio of fluorescently labeled and unlabeled cells permitted tracking of single cells over time, allowing us to study their migration during the early phases after implantation. Sequential images were aligned as accurately as possible based on SHG from extracellular matrix. A subset of labeled cells displayed a clear polarized morphology and moved in relation to the surrounding matrix, whereas numerous implanted cells remained in the same location over the duration of the experiment (Fig. 5 *A*). In a next step, we investigated the host vascular response after implantation by injecting fluorescently labeled dextran just before imaging. Host blood vessels were detected in the surrounding tissue during the first week after implantation, but they did not yet grow into the implant (data not shown). From 1 week after implantation on, host blood vessels started to invade into the construct (Fig. 5 *B*). To investigate when implanted cells started to differentiate into early osteoblasts, we implanted mPDC^FGF2^ expressing DsRed under the control of a collagen type I promoter fragment (Col1a1‐DsRed). Concurrent with the invasion of blood vessels into the constructs, implanted cells started expressing Col1a1‐DsRed (Fig. 5 *B*; Supporting Video 1). To further analyze the contribution of host and donor cells to bone formation, we implanted mPDC^FGF2^ expressing Col1a1‐DsRed into hosts ubiquitously expressing eGFP (actin‐GFP). At day 10 after implantation, a large number of DsRed‐positive cells was observed at the border of the construct, the site where new bone is first formed (Fig. 5 *C*; Supporting Video 2). Actin‐GFP host cells surrounded the implant, but only very few were observed within the construct. Finally, the constructs were explanted after MP‐IVM and quantification on histological sections showed that almost 20% of implanted cells had turned into Col1a1‐DsRed–positive cells (Fig. 5 *D*). Together, these data not only show the technical feasibility of MP‐IVM for bone tissue–engineered constructs in an imaging window, but also provide examples of how various reporters can be used to investigate the process of bone regeneration in vivo.

## Discussion

In the present study, we developed and validated a new ectopic imaging window that permits in vivo visualization of early processes in endochondral bone tissue–engineered implants using MP‐IVM. The major advantages are as follows: first, that this imaging window does not impair the bone forming process; second, that it is very adaptable in site and time of implantation; and third, that fluorescent reporters can be used to track specific processes such as angiogenesis or osteogenesis. Taken together, the newly developed imaging window provides an important tool to obtain more insight in the processes critical for bone regeneration and thereby stimulate clinical translation.

Intravital imaging has been a valuable tool in the in vivo study of osteoclast and osteocyte function,^31^, ^32^ osteoprogenitor lineage commitment and turnover,^16^ and has permitted sequential visualization of bone tissue–engineered constructs.^11^, ^12^ However, the setup mostly requires that the tissue of interest is surgically exposed every time when imaging is needed, and this procedure severely limits the frequency of visualization and may influence the observed processes. Imaging windows allow recurrent visualization and have already been used for observing dynamic processes in spinal cord,^33^ calvaria,^15^, ^19^ femur,^34^ and abdominal organs.^5^, ^14^ These imaging windows were however not intended or validated for the visualization of implanted engineered tissues. In the present study, we have validated an imaging window that allows repeated observation of an implanted bone tissue‐engineered construct in vivo. Moreover, it has several additional advantages. It is significantly less invasive than many larger imaging windows such as the dorsal skinfold chamber. Consequently, it causes less stretching and degradation of the surrounding skin and underlying muscle, which may indirectly affect the bone regeneration process. Furthermore, it can be placed in several locations, including at the shoulder, the back, and the skeletal muscles of the hind limb, which are common implantation locations for ectopic bone formation assays. These different locations also vary in the presence or lack of muscles and may provide information on the contribution of muscles to bone repair. One advantage of the dorsal skinfold chamber is that its large frame provides excellent stabilization during intravital imaging, but also in our model very little movement between sample and window occurred, leading to artifact‐free images. Finally, the newly developed imaging window is also very flexible with regard to timing between implantation of the construct and application of the window and thereby allows visualization of the early or rather late time points of bone regeneration depending on the research question.

The physiological relevance of findings obtained in an imaging window will largely depend on how well the processes of bone repair are preserved in the imaging window. We previously showed that the healing process in an orthotopic bone defect is very well mimicked in ectopic implantation assays and thus validated the ectopic assay as an appropriate model of bone regeneration.^(23,24)^ We now show that implanting a bone tissue‐engineered construct in the newly developed ectopic imaging window does not affect its bone‐forming capacity, making it a biologically relevant model. We have also found that not all imaging windows are suited for every MP‐IVM experiment: the physiology of the implanted tissue may be altered drastically already from early time points onward, as was observed through the lack of cartilage and bone formation by mPDC^FGF2^ in the dorsal skinfold window. At this moment we are unable to explain this lack of bone formation, but it is likely not due to absence of angiogenesis, which was present at day 10 after implantation (data not shown). Also the possibility of excessive mechanical pressure on the implant due to tension in the skinfold was ruled out using poly(lactic acid)/polycaprolactone (PCLA) spacers (data not shown). Other plausible explanations are a lower temperature in the tissues of the skinfold or the dependence of the implants on the presence of skeletal muscle to support bone formation, because skeletal muscle is lacking in the dorsal skinfold.

The asset of MP‐IVM is that one can use fluorescently labeled cell populations and reporters to investigate numerous physiological processes. We here show, as an example, the feasibility to analyze the early phases of bone formation in tissue‐engineered constructs in real time. Tracing of labeled cells following implantation revealed that they survive for several days and that only a subset of these cells show migratory behavior. In addition, intravenous injection of fluorescent dextran showed that vascular invasion of the construct requires several days. Moreover, both implanted and host cells can be transgenically labeled using lineage markers to study the contribution of various cell populations to bone regeneration over time. Additional reporters exist or are being developed, mainly in the field of tumor biology, to study processes such as cell survival,^35^ proliferation and hypoxia signaling,^36^ cellular redox potential,^37^ and even direct measurement of oxygen concentration in vivo.^38^ Combined with the high spatial resolution and relatively deep tissue penetration (up to 250 μm in bone) of multiphoton microscopy, these different reporters provide a valuable tool to dynamically investigate the physiology and interactions between implanted cells and the host microenvironment during bone regeneration in vivo, which is complementary to techniques such as μCT and histology.

In summary, we have developed an imaging window that permits sequential in vivo multiphoton imaging of bone tissue–engineered constructs with high temporal and spatial resolution over extended periods while preserving the biological processes. The establishment of this new model will facilitate further investigation into the spatiotemporal regulation of bone regeneration in a tissue‐engineered construct, aiding the development of new therapeutic strategies for improved healing of large bone defects.

## Disclosures

All authors state that they have no potential or definite conflicts of interest.

## Supporting information

Additional supporting information may be found in the online version of this article at the publisher's web‐site.

Supporting Table S1.

**Supporting Video 1**. 3D volume rendering of the image stack shown in Figure 5B. Shown are second‐harmonics generation (collagen matrix; blue), FITC‐labeled dextrans (blood vessels; green) and a collagen type I‐DsRed reporter (osteoblasts; red).

**Supporting Video 2**. 3D volume rendering of the image stack shown in Figure 5C. Shown are second‐harmonics generation (collagen matrix; blue), host cells expressing actin‐GFP (green), and a collagen type I‐DsRed reporter (donor osteoblasts; red).
