# Supplementary material for: An Ectopic Imaging Window for Intravital Imaging of Engineered Bone Tissue
Source: JBMR Plus. 2018 Jan 31;2(2):92–102. doi: 10.1002/jbm4.10028 (PMC6124161; doi:10.1002/jbm4.10028)
Supplement: Supplementary file 1 — Supporting Table S1. [file JBM4-2-92-s001.docx]

**Supporting Table 1.** Details of stainings performed and antibodies used for immunostaining

| **Staining** | **Details** |
| --- | --- |
| TRAP | Histochemical |
| CD31 | 1^st^ Ab: Rat anti-Mouse (MEC13.3, BD Pharmingen, USA) |
|  | 2^nd^ Ab: Biotin-conjugated Goat anti-Rat (559286, BD Pharmingen) |
|  | Substrate: DAB (S3000, DakoCytomation, Denmark) |
| COL2 | 1^st^ Ab: Mouse anti-Chicken (MAB8887, EMD Millipore, USA) |
|  | 2^nd^ Ab: FITC-conjugated Goat anti-Mouse (F0257, Sigma-Aldrich, Belgium) |
